# Role of Epithelial-Mesenchymal Transition in Retinal Pigment Epithelium Dysfunction

**DOI:** 10.3389/fcell.2020.00501

**Published:** 2020-06-25

**Authors:** Mi Zhou, Jasmine S. Geathers, Stephanie L. Grillo, Sarah R. Weber, Weiwei Wang, Yuanjun Zhao, Jeffrey M. Sundstrom

**Affiliations:** ^1^Department of Ophthalmology, Penn State College of Medicine, Hershey, PA, United States; ^2^Department of Medicine, The University of Texas Health Science Center at San Antonio, Houston, TX, United States

**Keywords:** RPE, dedifferentiation, RPE dysfunction, EMT, UPR, retinal degeneration

## Abstract

Retinal pigment epithelial (RPE) cells maintain the health and functional integrity of both photoreceptors and the choroidal vasculature. Loss of RPE differentiation has long been known to play a critical role in numerous retinal diseases, including inherited rod-cone degenerations, inherited macular degeneration, age-related macular degeneration, and proliferative vitreoretinopathy. Recent studies in post-mortem eyes have found upregulation of critical epithelial-mesenchymal transition (EMT) drivers such as TGF-β, Wnt, and Hippo. As RPE cells become less differentiated, they begin to exhibit the defining characteristics of mesenchymal cells, namely, the capacity to migrate and proliferate. A number of preclinical studies, including animal and cell culture experiments, also have shown that RPE cells undergo EMT. Taken together, these data suggest that RPE cells retain the reprogramming capacity to move along a continuum between polarized epithelial cells and mesenchymal cells. We propose that movement along this continuum toward a mesenchymal phenotype be defined as *RPE Dysfunction.* Potential mechanisms include impaired tight junctions, accumulation of misfolded proteins and dysregulation of several key pathways and molecules, such as TGF-β pathway, Wnt pathway, nicotinamide, microRNA 204/211 and extracellular vesicles. This review synthesizes the evidence implicating EMT of RPE cells in post-mortem eyes, animal studies, primary RPE, iPSC-RPE and ARPE-19 cell lines.

## Introduction

### The Epithelium to Mesenchyme Continuum

Epithelial cells and mesenchymal cells exhibit different characteristics and functions in the human body. Major hallmarks of terminally differentiated epithelial cells are expression of junctional complexes and apical-basal polarization. Epithelial cells reside on a basal membrane that separates them from other tissue components. In contrast to epithelial cells, mesenchymal cells are non-polarized cells with invasive and migratory behavior. Throughout embryogenesis, the capacity of cells to alternate between epithelial and mesenchymal states is vital for the development of the human body (Acloque et al., 2009; Kalluri and Weinberg, 2009). These processes are known as epithelial to mesenchymal transition (EMT) and mesenchymal to epithelial transition (MET). In healthy tissues, fully differentiated epithelial cells typically exert specific functions after development and are thought not to oscillate between two states. However, EMT can be activated under pathological circumstances, such as inflammation, wound healing, and carcinogenesis, enabling epithelial cells to obtain an enhanced migration ability and increase their production of extracellular matrix components.

**FIGURE 1:**
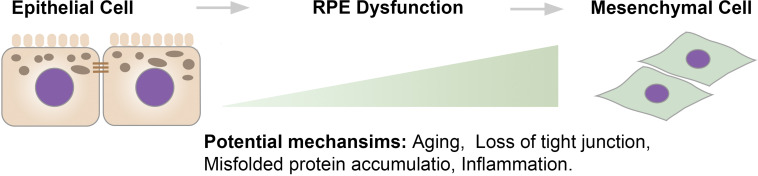
The epithelium to mesenchyme continuum. There is a continuum between epithelial cells and mesenchymal cells. Across the continuum, the transition starts from well-differentiated RPE cells that exhibit barrier integrity and cellular polarity to intermediate stages, where RPE cells become dedifferentiated and lose functions, to mesenchymal-like cells that exhibit an increased proliferative rate and migrate into the neuroretina.

Historically, EMT is classified into three subtypes: type I EMT occurs in the early stages of embryogenesis; type II EMT is associated with tissue regeneration and organ fibrosis; type III occurs in cancer cells and enables invasion and metastasis (Kalluri, 2009). Loss of epithelial markers, including zona-occludens-1 (ZO-1), E-cadherin, and cytokeratin, and gain of expression of mesenchymal drivers, including vimentin, N-cadherin, and fibronectin, are encompassed in the classical definition of EMT. However, this definition remains heavily debated and is thought to be oversimplified. Indeed, gain of invasive and migratory abilities is not necessarily accompanied by the complete loss of epithelial traits. Moreover, Huang et al. (2012, 2013) showed that ovarian cancer cells are heterogeneous, as some cells lose E-cadherin but do not gain N-cadherin. As such, it has been proposed that EMT and MET exist on a continuum, and an intermediate phenotype exists (Nieto et al., 2016). Across this continuum, factors that drive the transition from epithelial to mesenchymal remain to be determined. Herein, the transition from fully differentiated epithelial cells toward mesenchymal cells (including the intermediate states) is defined as RPE dysfunction. Potential mechanisms involved in RPE dysfunction may include aging, loss of tight junctions, accumulation of misfolded protein, and inflammation (Figure 1).

### Retinal Pigment Epithelial (RPE) Cells

The RPE form a single layer of highly polarized cells juxtaposed between the photoreceptors and choroid. There are approximately 3.5 × 10^6^ RPE cells in each adult human eye. Classically, RPE cells are thought to be terminally differentiated throughout life (Panda-Jonas et al., 1996). Several signaling pathways have been reported to be involved in RPE differentiation, including Sonic hedgehog (Shh), Wnt/β-catenin, and Notch (Perron et al., 2003; Burke, 2008; Schouwey et al., 2011; Amirpour et al., 2012). MicroRNAs (miRNAs) also play a role; previous studies have shown that miRNA204/211 are critical for maintaining RPE differentiation (Wang et al., 2010; Adijanto et al., 2012). RPE cells display morphological polarity, with apically located microvilli, tight junctions, and melanosomes, and basally located nuclei and basal infoldings. Melanin pigment granules in RPE cells absorb light, contributing to visual function and protecting against photo-oxidative stress (Sundelin et al., 2001). Basally, RPE cells attach to Bruch’s membrane, which consists of a mixture of collagen type IV, laminin, and fibronectin that is similar to other basement membranes and functions to separate the RPE from the choriocapillaris (Libby et al., 2000).

**FIGURE 2:**
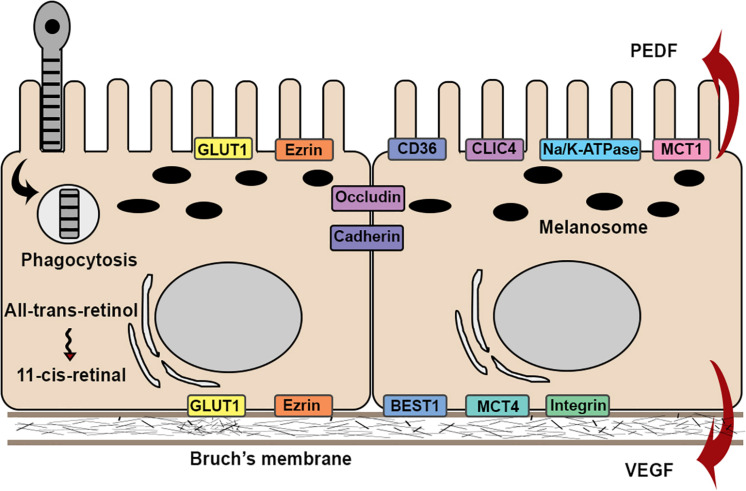
Schematic illustration of the structures and functions in RPE cell. RPE cells attach to Bruch’s membrane in a single monolayer with apical microvilli and melanosomes, and basal nuclei. Barrier function is maintained by expression of occludin and cadherins. RPE cells exhibit physiological functions, such as phagocytosis and visual cycles. RPE cells exhibit functional polarity by polarized secretion of proteins and apical-basal distribution of membrane proteins.

The structure of RPE cells is optimized for their many functions. Proper RPE function requires specific polarized distribution of transmembrane proteins. For example, Na^+^/K^+^-ATPases (Wimmers et al., 2007), chloride intracellular channel 4 (CLIC4) (Wimmers et al., 2007), mannose receptors (Tarnowski et al., 1988), and proton-coupled monocarboxyate transporters 1 (MCT1) (Deora et al., 2005) are restricted to the apical aspect of RPE cells. CD36 that functions in phagocytosis is also apically located (Ryeom et al., 1996). In contrast, integrins, MCT3 (Yoon et al., 1997), and Bestrophin-1 (Milenkovic et al., 2011), a chloride anion channel, are located basally. In addition to the polarized distribution of membrane proteins, RPE cells secrete proteins in a polarized manner. Vascular endothelial growth factor (VEGF) is primarily secreted in the basal direction to promote the growth of the choroidal vasculature, whereas pigment epithelium-derived factor (PEDF), an angiogenic inhibitor, is apically secreted (Bhutto et al., 2006). Some proteins lack a polarized distribution, including ezrin (Bonilha et al., 1999) and Glucose transporter (GLUT) 1 (Senanayake et al., 2006), which exist on both apical and basal aspects of the cell (Figure 2).

## Clinical and Preclinical Evidence of EMT in RPE Cells

### Overview

**FIGURE 3:**
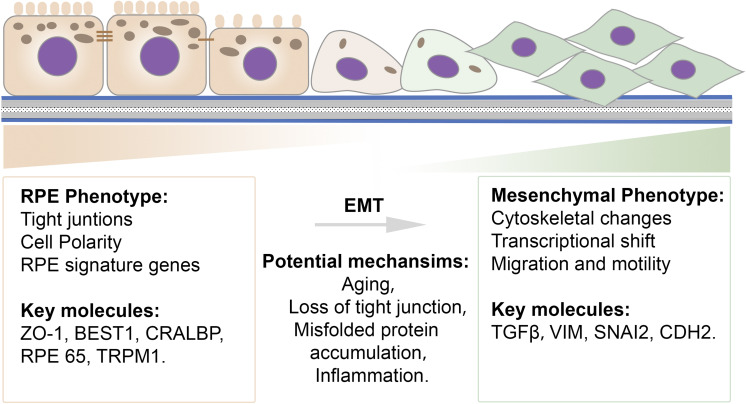
Schematic illustration of the transition spectrum in RPE cells. Common epithelial and mesenchymal cell features and key molecules are listed. RPE cells exhibit a well-differentiated epithelial phenotype that is characterized by integrated tight junctions, high polarization, and proper RPE signature gene expression. A mesenchymal-like phenotype exhibits cytoskeletal changes, a transcriptional shift, and an increased ability for migration. Potential mechanisms contributing to EMT in RPE cells include aging, loss of tight junctions, misfolded protein accumulation and inflammation.

Together, photoreceptors, retinal pigment epithelial cells (RPE), and the choroid form a functional unit that is required for proper visual function. RPE cells are the central component of this unit, as they maintain the health and functional integrity of both photoreceptors and the choroid. RPE dysfunction is often an initiating or early factor in retinal disease, manifesting as a loss of RPE barrier function, disrupted RPE polarization, and downregulated microRNA-204/211 expression. Emerging evidence shows that RPE cells become less differentiated and subsequently undergo EMT with upregulated mesenchymal cell markers and enhanced migration ability in several degenerative retinal diseases (Tamiya and Kaplan, 2016; Ghosh et al., 2018; Wu et al., 2018; Figure 3). Clinical evidence suggesting that RPE cells undergo EMT in macular degenerations and proliferative vitreoretinopathy (PVR), as well as potential underlying mechanisms, are discussed in the following sections.

### Hyperreflective Foci

Hyperreflective foci (HRF) are well-circumscribed lesions with equal or greater reflectivity than the RPE band that spread over various retinal and choroidal layers in spectral domain optical coherence tomography (SD-OCT) images (Liu S. et al., 2019; Roy et al., 2019). Numerous clinical studies have shown that retinal HRF appear in optical coherence tomography OCT images in retinal diseases, including age-related macular degeneration (AMD), inherited rod-cone degenerations, and inherited macular degenerations (Kuroda et al., 2014; Piri et al., 2015; Miura et al., 2017). HRF in choroid have been reported in Stargardts disease (Piri et al., 2015) and diabetic macular edema (DME) (Roy et al., 2019). The cellular origins of HRF were initially hypothesized to be migrated RPE cells, macrophages, or hard exudates. Emerging evidence confirms that a portion of HRF are RPE cells (Chen et al., 2016; Miura et al., 2017). A previous study investigated the origin of HRF by combining polarimetry with auto-fluorescence imaging, enabling differentiation between RPE cells, inflammatory cells, and hard exudates. The results revealed that, in early stages of AMD, a portion of HRF are likely secondary to RPE migration (Miura et al., 2017). Moreover, histopathological studies confirmed that HRF represent cells of RPE origin, substantiating the idea that RPE cells have the capacity to migrate into neuroretina (Chen et al., 2016).

In numerous retinal degenerative diseases, HRF were found to correlate with disease stage. In AMD, HRF were found to correlate with pigmentary changes on fundus imaging and were seen with increased frequency in advanced forms of AMD, including geographic atrophy and choroidal neovascularization (CNV) (Christenbury et al., 2013). In the latter case, anti-VEGF treatment has been shown to significantly decrease the amount of HRF (Abri Aghdam et al., 2015). In addition to AMD, HRF appear to hold clinical significance in inherited retinal degenerations. For example, in Stargardts disease, an inherited macular degeneration caused by a single mutation in the *ABCA4* gene, the appearance of HRF was found to correlate strongly with poor visual acuity, decreased central macular thickness, and increased disease duration (Piri et al., 2015). In retinitis pigmentosa, HRF were observed more often in the inner nuclear layer in early stages, whereas they were more frequently observed in the outer nuclear layer in later stages, indicating that HRF location correlates with disease stage (Kuroda et al., 2014). Thus, HRF may be viewed as a predictive marker for disease progression, and migratory RPE cells can be seen as a sign of poor prognosis.

### Evidence of EMT in Age-Related Macular Degeneration (AMD)

#### Clinical Evidence

Age-related macular degeneration is a leading cause of irreversible vision loss, accounting for 13.4 million cases worldwide (Friedman et al., 2004). The primary pathology of AMD occurs at the level of the RPE cells. As RPE cells become atrophic, the hallmark clinical lesions of AMD ensue; sub-RPE lipoprotein deposits, known as drusen and drusenoid deposits, and RPE pigment disruption become visible within the macula. Most AMD begins after the age of 55 as the “dry” form of macular degeneration. In 10–15% of AMD cases, it progresses to the “wet” form of the disease, which is defined by CNV. Drusen deposits typically form between the RPE and Bruch’s membrane, which can create a mechanical tension that negatively impacts cell-cell contacts. A prior study showed that the volume of drusenoid pigment epithelium detachment (PED) was inversely correlated with visual acuity (Balaratnasingam et al., 2016). An increase in drusenoid PED size promotes the disintegration of RPE layer and facilitates RPE migration (Balaratnasingam et al., 2016). Likewise, in wet AMD patients, increased area of serous PEDs is strongly associated with RPE layer disruption (Miura et al., 2019). In CNV, abnormal vessel growth and retinal hole formation directly disrupt cell-cell contact between RPE cells.

The Age-Related Eye Disease Study (AREDS) identified two clinical risk factors for disease progression, namely, drusen burden and the presence of pigment abnormalities (Ferris et al., 2005). Patients with either large drusen or macular pigment are at a 13 and 12.5% risk of developing advanced AMD, respectively. Interestingly, when the drusen burden is large and pigment is present, the risk of progression increases synergistically to 47.3%. However, the molecular mechanisms that drive RPE dysfunction and lead to pigment accumulation in macular degeneration remain to be determined.

**FIGURE 4:**
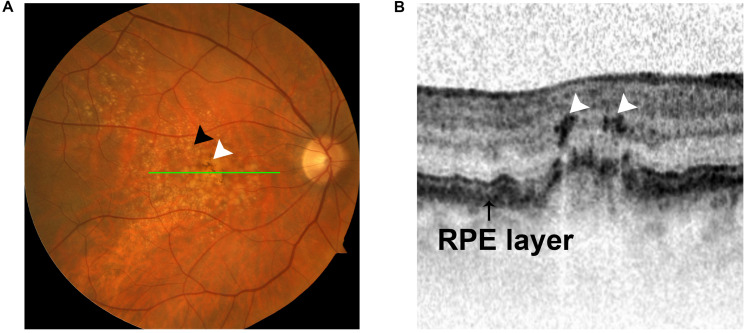
Intraretinal hyperreflective foci (HRF) appear in OCT images in intermediate AMD. **(A)** Fundus photograph showing two hallmarks of intermediate AMD: sub-RPE deposits (black arrow) and pigment changes (white arrow). **(B)** OCT image demonstrating the presence of intraretinal HRF (white arrows) in intermediate AMD. The HRF correlate with pigment on funduscopic imaging (green line).

**FIGURE 5:**
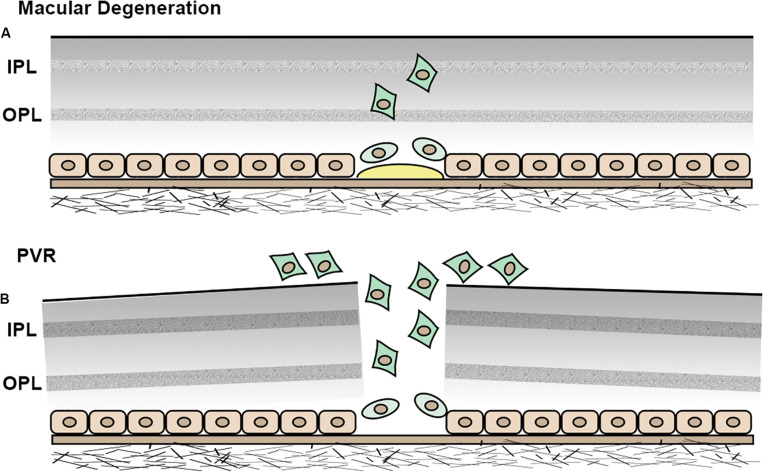
Schematic illustration of EMT in RPE cells. **(A)** In macular degeneration, RPE cells become less differentiated, undergo EMT and migrate into neuroretina. **(B)** In PVR, RPE cells undergo EMT and migrate into the epiretinal area.

As mentioned in the previous section, HRF are observed on OCT in AMD patients and correlate with abnormal pigment on fundus imaging, suggesting that HRF and pigmentary changes represent RPE cells that have migrated into the neuroretina (Folgar et al., 2012; Christenbury et al., 2013; Figure 4). This concept is substantiated by studies demonstrating EMT of RPE cells in AMD. Ghosh et al. (2018) demonstrated upregulated expression of the EMT transcriptional markers vimentin and Snail1 and downregulated expression of E-cadherin in RPE cells in post-mortem human dry AMD eyes relative to age-matched controls, suggesting that RPE cells in dry AMD patients had undergone EMT. Hirasawa et al. (2011) showed that Snail co-localized with RPE65-positive cells in 11 human CNV eyes. Additionally, this study found that Snail- and α-SMA-double-positive RPE cells were strongly associated with RPE fibrotic changes, indicating that RPE cells undergo epithelial-myofibroblast transition, and that this transition leads to retinal fibrosis, an end-stage manifestation of wet AMD (Ishikawa et al., 2016a; Roberts et al., 2016; Figure 5A).

Here, we hypothesize that pigment abnormalities represent RPE cells that have undergone EMT and migrated into the neuroretina. A contradiction to this hypothesis is that RPE cells should, in theory, become less pigmented with EMT, and, therefore, would not result in a pigmented macula. However, it is possible that migrating RPE cells become less differentiated, but remain pigmented to, perhaps, a lesser degree. It is also possible that macular hyperpigmentation results from a cluster of RPE cells that have migrated to this area in order to make up for the reduction in pigmentation. Another possibility is that migrating RPE cells undergo MET in the neuroretina and become more pigmented. Further studies should focus on the molecular mechanisms that drive RPE dysfunction and lead to pigment accumulation in macular degeneration.

#### Preclinical Evidence

Published studies using animal models of AMD have shown that proteins, such as PCG-1-alpha, MRTF-A and PTEN, are involved in the EMT of RPE cells, as inhibiting their activity attenuated the severity of disease progression (Kim et al., 2008; Kobayashi et al., 2019; Rosales et al., 2019). Interestingly, Ishikawa et al., 2016b found that *α*β-crystallin, a protein linked to AMD pathophysiology, was found to modulate EMT through SNAIL and SLUG. They showed that suppressing *α*β-crystallin results in the inhibition of EMT development in RPE cells (Ishikawa et al., 2016b).

### Evidence of EMT in Proliferative Vitreoretinopathy (PVR)

#### Clinical Evidence

Proliferative vitreoretinopathy is a scarring process that occurs following the treatment of rhegmatogenous retinal detachment. The incidence of post-operative PVR is estimated to be 5–10% (Bonnet et al., 1996; Rodriguez de la Rua et al., 2003). PVR is initiated by a retinal break, which is followed by persistent inflammation and wound healing. RPE cells are believed to contribute to the healing process by undergoing EMT and migrating to the epiretinal area (Yamashita et al., 1986; Morino et al., 1990; Casaroli-Marano et al., 1999). The resultant formation and contraction of PVR membranes ultimately lead to retinal folds and loss of vision (Figure 5B).

Several studies have shown that cytokeratin, an RPE cell marker, co-localized with vimentin in PVR membranes (Yamashita et al., 1986; Morino et al., 1990; Casaroli-Marano et al., 1999). Additionally, Feist et al. (2014) showed that, in human PVR membranes, cells positive for cytokeratin were co-expressed with α-SMA. By counting the cells positive for both cytokeratin and α-SMA, the study showed that the majority of myofibroblasts in human PVR membranes originated from RPE cells, suggesting that RPE cells are capable of undergoing EMT and migrating into neuroretina, and that this process plays a major role in the pathogenesis of PVR.

#### Preclinical Evidence

A number of PVR animal studies have shown that RPE cells undergo EMT when PVR is induced (Saika et al., 2004; Nagasaki et al., 2016; Yoo et al., 2017; Zhang et al., 2017). Following retinal detachment, one study found that RPE cells would stain positive for alpha-SMA in wild-type mice, but RPE cells in Smad3-null mice would be negative, indicating that EMT is attenuated when Smad3 is absent (Saika et al., 2004). The same investigation also found that Smad3-null mice had decreased residual subretinal fibrosis. Additionally, PVR in mice induced by dispase injection has been shown to increase retinal alpha-SMA-positive cells (Yoo et al., 2017). Together, these *in vivo* studies suggest that RPE cells undergo EMT following PVR, whereby suppressing the EMT process can greatly reduce the severity of PVR.

### Evidence of EMT in Inherited Retinal Degenerations (IRDs)

#### Clinical Evidence

A subtype of Best disease, autosomal dominant vitreoretinochoroidopathy (ADVIRC) is a chorioretinal degeneration caused by a mutation in the Bestrophin-1 (*BEST1*) gene, and evidence suggests that EMT of RPE cells plays a role in its pathogenesis. In a study of ADVIRC post-mortem human eyes, Goldberg et al. demonstrated minimal expression of TGF-β within the RPE cell monolayer (Goldberg et al., 2018). In contrast, RPE cells that had migrated into the neuroretina have been characterized as having downregulated RPE-65 and upregulated expression of TGF-β, suggesting that RPE cells become dedifferentiated once they start to migrate (Goldberg et al., 2018). In inherited retinitis pigmentosa, proliferative and displaced RPE cells have been observed in regions up to the internal limiting membrane in several patient samples (Szamier and Berson, 1977; Fox, 1981; Flannery et al., 1989; Li et al., 1995). One study showed that RPE cells in a spared region of retina had apically displaced nuclei (loss of polarization) and abundant melanolysosomes, whereas RPE cells in areas of more severe disease were flattened and depigmented (Szamier and Berson, 1977). In photic maculopathy, transmission electronic microscopy (TEM) indicated that RPE cells were displaced and proliferative with depigmentation, loss of infoldings, and irregular shape (Tso, 1973). Another study demonstrated RPE cell proliferation in retinal detachment and found that new cells did not display typical RPE cell polarity (Anderson et al., 1981). In choroideremia and chorioretinal atrophies, regions of proliferating and attenuated RPE cells were observed, with abrupt transitions in between them (Curcio et al., 2000; Jonasson et al., 2007; MacDonald et al., 2009).

## Mechanisms of EMT in RPE Cells

### Overview

The molecular mechanisms that drive RPE dysfunction and lead to retinal degeneration remain to be determined. Emerging evidence suggests that the impairment of tight junctions and accumulation of misfolded proteins drive EMT in RPE cells. Moreover, upregulation of TGF-β and Wnt pathway appears to to play a critical role in RPE de-differentiation and promoting EMT. In contrast, Nicotinamide and microRNA 204/211 have been shown to enhance the RPE phenotype and prevent EMT in multiple RPE cell model systems. Extracellular vesicles that have been shown to regulate EMT in numerous other tissues are also discussed in the following sections.

### Roles of Junctional Proteins in EMT

Cell-cell contact is critical for maintaining an epithelial phenotype. The RPE acts as part of the outer blood-retina barrier by way of tight junctions (TJs) and adherens junctions (AJs) between neighboring RPE cells (Foulds et al., 1980). At a molecular level, the major proteins that constitute TJs in RPE cells are occludin and claudins, while cadherins serve as the major AJ proteins (Erickson et al., 2007; Tash et al., 2012). The extracellular domains of these molecules form an intact blood-retinal barrier that results in a high transepithelial resistance.

A prior study showed that RPE cells in the center of cultured sheets maintained a well-differentiated phenotype with proper expression of RPE signature genes and pigment, whereas RPE cells at the edges of the sheet lost epithelial morphology and were less pigmented (Tamiya et al., 2010). Moreover, this study showed upregulated expression of vimentin and N-cadherin in RPE cells at the edges, suggesting that loss of cell-cell contact leads to RPE cell de-differentiation and facilitates EMT of RPE cells. Georgiadis et al. (2010) performed *in vivo* studies in C57/Bl6 mice, utilizing shRNA to target and disrupt junctional proteins, like ZO-1, which induced RPE cell proliferation and hyperpigmentation of the retina (Georgiadis et al., 2010). The RPE cells were found to be undergoing EMT, whereby the absence of ZO-1 induced the expression of key EMT markers and reduced the expression of epithelial markers in the affected RPE cells (Georgiadis et al., 2010). These data show that the loss of the tight junction protein causes RPE cell dedifferentiation and induces EMT.

**FIGURE 6:**
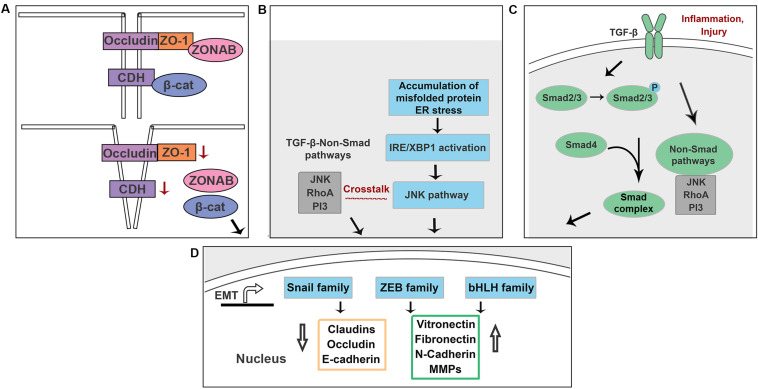
Schematic illustration of the potential mechanisms underlying EMT in RPE cells. **(A)** Loss of ZO-1 and E-cadherin lead to a release of ZONAB and β-catenin. **(B)** Accumulation of misfolded proteins activate NK/p38-MAPK pathway that crosstalks with TGF-β-induced non-Smad pathways in an IRE-dependent manner. **(C)** Increased production of TGF-β is associated with inflammation and injury in RPE cells. **(D)** Above signals promote EMT by upregulating EMT drivers including Snail, Zeb and bHLH family.

The mechanism by which TJs and AJs maintain an epithelial phenotype involves sequestration of EMT signals within their complexes. The junctional protein ZO-1 binds to ZO-1–associated Y-box factor (ZONAB), a transcription factor that is able to upregulate cell proliferation rate by regulating nuclear expression of cyclin-dependent kinase 4 (CDK4) (Ikenouchi et al., 2003; Gonzalez-Mariscal et al., 2014). Loss of ZO-1 results in a release of ZONAB into the cytoplasm, enabling a subsequent translocation of ZONAB (Erickson et al., 2007). ZONAB then binds to the transcriptional factor for CDK4, increasing expression of CDK4 and promoting cell proliferation. Similarly, E-cadherin sequesters β-catenin on the cell membrane, and downregulation of E-cadherin leads to a release of β-catenin into the cytoplasm. β-catenin subsequently translocates into the nucleus and activates promoters of EMT and proliferation, including Snail and Cyclin D1 (Gonzalez and Medici, 2014; Figure 6A). The Hippo-YAP pathway, an important regulator of RPE cell differentiation, depends strongly on cell junction complexes. TJ and AJ complexes inhibit YAP/TAZ translocation (two effectors of the Hippo pathway) into the nucleus. Loss of TJs or AJs enables activation of YAP/TAZ. Activated YAP/TAZ bind to TEAD and upregulate ZEB1, promoting EMT (Lei et al., 2008). A previous study showed that knockdown of YAP in primary mouse RPE cells downregulated MITF expression and upregulated ZEB1 expression, indicating that loss of YAP attenuates RPE differentiation and induces RPE cells to undergo EMT (Liu et al., 2010). Thus, RPE cell-cell junctions are critical regulators of EMT.

### Roles of Misfolded Proteins in EMT

Emerging evidence suggests that the unfolded protein response (UPR) and EMT signaling interact in several different organs (Zhong et al., 2011; Lenna and Trojanowska, 2012; Pang et al., 2016; Santamaria et al., 2019; Figure 6B). Mutations in *BEST1* cause its mislocation and results in retinitis pigmentosa and other retinal dystrophies (Johnson et al., 2014, 2017). Milenkovic et al. (2018) showed that recessive mutations in *BEST1* activate the UPR with upregulated expression of XBP1. Morton et al. showed that in ADVIRC patient, a chorioretinal degeneration caused by a mutation in the *BEST1* gene, RPE cells that had migrated into the neuroretina have been characterized as having downregulated RPE-65 and upregulated expression of TGF-β (Goldberg et al., 2018). A previous study showed that accumulation of intracellular amyloid-β attenuates TJs of RPE cells by downregulating occludin and claudin-1 proteins, supporting the idea that accumulation of abnormal proteins in RPE cells attenuates RPE differentiation (Park et al., 2014). Crystallin is a protein expressed in the eye and is classified into three types: α, β, and γ crystallins. βA3/A1-crystallin localizes to the lysosome and plays a critical role in the clearance functions of lysosomes, including phagocytosis and autophagy (Zigler and Sinha, 2015). A prior study demonstrated that the expression level of βA3/A1-Crystallin in polarized primary human RPE cells is 23.75-fold higher than that of non-polarized primary human RPE cells (Ghosh et al., 2018). Moreover, knockdown of βA3/A1-Crystallin in human and murine RPE cells results in upregulated expression of Snail and vimentin and an enhanced migration ability (Ghosh et al., 2018). This evidence suggests a connection between the protein misfolding and EMT in RPE cells. Doyne honeycomb macular dystrophy (DHC) results from an R345W mutation in Fibulin-3 (Marmorstein, 2004; Narendran et al., 2005), an extracellular matrix protein and downstream target of HTRA1 (Lin et al., 2018). The R345W mutation causes Fibulin-3 misfolding, poor Fibulin-3 secretion, and activation of the UPR (Marmorstein et al., 2002; Hulleman and Kelly, 2015). In our lab, we found that overexpression of R345W-Fibulin-3 in primary human RPE cells activates the UPR via the IRE1α/XBP1 pathway, attenuates RPE differentiation and facilitates EMT in RPE cells (Zhou et al., 2020; accepted in Frontiers in Cell and Developmental Biology). Taken together, these data suggest that the accumulation of misfolded proteins in RPE cells contributes significantly to EMT and likely accelerates the disease process in patients who have inherited macular degenerations.

UPR activation involves several mechanisms aimed at reducing the load of aberrant protein accumulation, including attenuated protein translation to avoid worsening the accumulation, increased transcription of endoplasmic reticulum (ER) chaperones to aid in the folding process, and an increase in ER-associated degradation (ERAD). Three ER transmembrane sensors, IRE1α/XBP1, PERK, and ATF6, regulate the UPR and determine the appropriate adaptive response, directing the cell to proliferate, change shape, or undergo apoptosis (Lin et al., 2007; Walter and Ron, 2011). Several studies have shown that the UPR and TGF-β-induced EMT signaling pathways interact at the level of c-Jun N-terminal kinase and p38 mitogen-activated protein kinase (JNK/p38-MAPK) in an IRE1-dependent manner (Urano et al., 2000; Santibanez, 2006; Liu Z. et al., 2019). The activation of IRE1α/XBP1 signaling promotes EMT by upregulating JNK and EMT drivers, including Snail and Zeb family members, in different organs and tissues, including the breast, lung, and liver (Li et al., 2015; Mo et al., 2015; Cuevas et al., 2017; Liu Z. et al., 2019). Future studies should focus on the mechanisms by which ER stress regulates EMT in RPE cells.

### Key Pathways and Molecules

#### TGF-β Pathway

TGF-β acts as an anti-inflammatory cytokine; increased production of TGF-β is associated with injury and inflammation. TGF-β signaling induces EMT by activating either Smad or non-Smad pathways (Xu et al., 2009). In the Smad-dependent pathway, phosphorylation of TGF-β receptor (Type I and Type II) recruits Smad2 and Smad3 (Valcourt et al., 2005). The phosphorylation of Smad2 and Smad3, then recruits Smad4 and facilitates the formation of the Smad-complex. The Smad-complex translocates into the nucleus and binds regulatory elements that in turn induce the transcription of several key genes associated with EMT (Gonzalez and Medici, 2014). In the Smad-independent pathway, TGF-β cross-talks with the JNK/p38-MAPK pathway to regulate EMT in an IRE1α-dependent manner (Engel et al., 1999; Zhou et al., 2004; Figure 6C).

Previous studies have shown that the amount of TGF-β in the vitreous from eyes with PVR is three times higher than that of eyes without intraocular fibrosis (Connor et al., 1989; Kita et al., 2008). Subconfluent culturing of primary human RPE cells, which mimics a wound stimulus, has been shown to result in acquisition of a mesenchymal phenotype by activating the TGF-β pathway (Radeke et al., 2015). Treatment with TGF-β and TNF-α has been found to accelerate EMT in adult human RPE stem cell –derived RPE cell cultures (Boles et al., 2020). Blockage of TGF-β and FGF/MAPK pathways has been shown to markedly promote RPE differentiation efficiency during induced pluripotent stem cell (hiPSC)-derived RPE cell culture (Kuroda et al., 2019).

Most of the *in vivo* investigations of EMT mechanisms in RPE cells involve genetic (Saika et al., 2004; Wu et al., 2019) or pharmacological (Ishikawa et al., 2015; Yoo et al., 2017; Zhang et al., 2017) manipulation of the TGF-β pathway. Ishikawa et al. (2015) used a PVR rabbit model to pharmacologically inhibit transforming growth factor-β2(TGF-β2)-induced EMT of RPE cells using Resveratrol, a compound that deacetylates SMAD4 (Ishikawa et al., 2015). To determine the effect of notch inhibition on TGF-β1-induced EMT, Zhang et al. (2017) injected ARPE-19 cells that had been pre-incubated with a γ-secretase Notch inhibitor (LY411575) in a PVR mouse model, and found EMT was attenuated when Notch signaling was inhibited (Zhang et al., 2017). In a PVR *in vivo* rat model, miR-194 decreased the ZEB1 protein. ZEB1 can synergize with SMAD and lead to TGF-β-dependent gene transcription; therefore, suppression of this process repressed EMT in RPE cells (Cui et al., 2019). The absence of Galactin-1, a galactoside-binding lectin family protein which modifies the TGF-β pathway as well as others, in a knockout mouse following CNV resulted in reduced CNV severity, level of subretinal fibrosis and expression of EMT-related markers in RPE cells (Wu et al., 2019). Taken together, these results show the importance of the different components of the TGF-β pathway in the RPE-EMT process (Figure 6D).

#### Wnt Pathway

Wnt-β-catenin is another well-characterized pathway that mediates EMT in the eye. A previous study showed that, in mouse eyes, laser photocoagulation activated the Wnt/β-catenin pathway and facilitated RPE proliferation and EMT (Han et al., 2015). In ARPE-19 cells, EMT was promoted by the overexpression of β-Catenin and was blocked by a Wnt Inhibitor (XAV939) (Chen et al., 2012). Light exposure has also been shown to induce EMT in RPE cells by activating the Wnt/β-catenin pathway (Iriyama et al., 2008).

β-catenin is a key element in the Wnt signaling pathway. Without the activation of Wnt signaling, β-catenin is sequestered by a complex of glycogen synthase kinase-3 beta (GSK-3β) and Axin. Activation of Wnt signaling leads to a release of β-catenin from the complex, enabling the subsequent translocation of β-catenin. Nuclear β-catenin then binds to the transcriptional factors for Snails, leading to increased expression levels of Snail and promotion of EMT.

#### Nicotinamide

Nicotinamide (NAM), a vitamin B3 derivative, has both antioxidant and anti-inflammatory properties. NAM has been shown to enhance the RPE phenotype and prevent EMT in multiple RPE cell model systems (Saini et al., 2017; Meng et al., 2018; Hazim et al., 2019; Boles et al., 2020). ARPE-19 cell line is not typically thought to be well differentiated but does offer relative convenience and consistency. NAM has been shown to rapidly promote ARPE-19 cell differentiation (Hazim et al., 2019). In human adult RPESC-RPE, NAM prevents and reverses RPE EMT that is induced by TGF-β and TNF-α treatment (Boles et al., 2020). In a human iPSC model of AMD, NAM ameliorates disease phenotype by inhibiting drusen proteins and inflammatory and complement factors (Saini et al., 2017). Potential mechanisms by which NAM promoted RPE cell survival and differentiation include inhibiting Rho-associated protein kinase (ROCK) and casein kinase 1 (CK1) (Meng et al., 2018).

#### microRNA-204/211

miRNA-204/211 plays a critical role in RPE cell differentiation. Prior studies demonstrated that the TGF-β receptor is a direct target of miRNA-204/211 in RPE cells. Transient receptor potential cation channel (TRPM)1 and TRPM3 are two signature genes of RPE cells. miRNA-204 resides in the sixth intron of TRPM3, and miR-211 resides in the sixth intron of TRPM1. miRNA-204/211 and TRPM3/1 co-translate in RPE cells (Adijanto et al., 2012). miRNA-204 and miRNA-211 are highly expressed in fully differentiated RPE cells, allowing maintenance of RPE terminal differentiation (Wang et al., 2010). In contrast, both TRPM1/3 and miRNA-211/204 are downregulated in dedifferentiated RPE cells (Wang et al., 2010). Further, anti-miRNA-204/211 leads to elevations in the levels of several EMT transcriptional factors, including TGFBR2, JNK, SNAIL1, SNAIL2, Smad3, and Smad4. Together, these data demonstrate the importance of miRNA-204/211 in preventing EMT in RPE cells.

#### Extracellular Vesicles

Extracellular vesicles (EVs) play a critical role in cell-cell communication, modulate cellular differentiation, and promote aggregate formation (Yuyama et al., 2008; Alvarez-Erviti et al., 2011). EVs can originate from either multivesicular bodies (MVBs) or from the plasma membrane. They have recently been shown to be a major constituent of the vitreous body in the eye (Zhao et al., 2018). Prior studies have shown that alterations in EV cargo are representative of the phenotypic status of their parental cells (Vella, 2014; Kim et al., 2016). EVs contribute to the regulation of EMT and promote cancer metastasis in numerous tissues, including the lungs, breasts, liver and brain (Vella, 2014; Kim et al., 2016; Chen et al., 2017; van de Vlekkert et al., 2019). Chen et al. (2017) showed that EVs derived from p85α^–/–^ fibroblasts that possess greater mesenchymal features promoted breast cancer cells migration and invasion compared with EVs from WT fibroblasts (Chen et al., 2017). van de Vlekkert et al. (2019) showed that myofibroblast-derived EVs are sufficient to induce normal fibroblasts to become myofibroblasts that possess greater mesenchymal features, by upregulating TGF-β pathways and EMT drivers. Although the specific role of EVs in mediating RPE cell EMT remains to be determined, they appear to be involved in numerous mechanisms relating to EMT in RPE cells.

## Therapeutic Implications

Many preclinical studies using promising therapeutic interventions have been shown to effectively rescue RPE cells from EMT, including TGF- β receptor inhibitors, peroxisome proliferator-activated receptor (PPAR)-γ agonists, retinoic acid receptor-γ (RAR-γ), and anti-inflammation agents. The TGF-β receptor I inhibitor LY-364947 has been shown to reduce RPE transdifferentiation *in vitro* and prevent PVR development in a rabbit model (Nassar et al., 2014). By injecting a negative TGF-β receptor II, the severity of PVR was significantly attenuated in the rabbit eye (Yamada et al., 2007). PPAR-γ and RAR-γ possess anti-inflammatory properties and regulate EMT as a result of their attenuation of TGF-β actions. In rats, PPAR-γ agonists have been shown to attenuate fibrosis in several organs, including the heart, liver, lungs, and kidney, by inhibiting the TGF-β pathway (Galli et al., 2002; Aoki et al., 2009; Higashi et al., 2010; Elrashidy et al., 2012). Previous studies showed that the PPAR-γ agonists Troglitazone and Pioglitazone prevent TGF-β2-induced EMT in RPE cells by inhibiting Smad phosphorylation (Cheng et al., 2008; Hatanaka et al., 2012). Similar to PPAR-γ, RAR-γ also plays a role in mediating fibrosis in several organs. A prior study showed that an RAR-γ agonist inhibits the development of subretinal fibrosis in mice by inhibiting the TGF-β pathway (Kimura et al., 2015). Bone morphogenetic proteins (BMPs) are pluripotent growth factors which have anti-fibrotic activity (Yao et al., 2019). Injections of BMP7 in a rabbit PVR model maintained RPE cell phenotypes and prevented TGF-β2-induced EMT, migration and gel contraction (Yao et al., 2019). Anti-inflammatory agents, including Bortezomib, a proteasome inhibitor that regulates the NF-κB pathway, and resveratrol, a polyphenol phytoalexin and heavy chain-hyaluronan/pentraxin3, inhibit EMT in RPE cells and prevent PVR development by downregulating the TGF-β pathway (Ishikawa et al., 2015; He et al., 2017; Moon et al., 2017). Thus, many possible avenues exist for the application of therapeutics aiming to alleviate EMT of RPE cells, potentially preventing vision loss in retinal disease.

## Conclusion

Emerging evidence suggests that RPE cells undergo EMT and migrate into the neuroretina in certain pathological conditions, manifesting clinically as HRF in OCT that correlate with pigmentary changes on funduscopy. The data summarized here indicate that EMT of RPE cells is a significant predictor for disease prognosis. We summarized recent advances and potential mechanisms underlying this process. These advances may help clarify the role of EMT in retinal disease states and point to avenues that can be exploited for the development of new therapeutic targets.

## Author Contributions

MZ, JG, SG, SW, WW, YZ, and JS contributed in writing the review.

## Conflict of Interest

The authors declare that the research was conducted in the absence of any commercial or financial relationships that could be construed as a potential conflict of interest.
